# The impacts of host association and perturbation on symbiont fitness

**DOI:** 10.1007/s13199-024-00984-6

**Published:** 2024-04-02

**Authors:** Kim L. Hoang, Roberto Salguero-Gómez, Victoria L. Pike, Kayla C. King

**Affiliations:** 1 Department of Biology, University of Oxford, Oxford, UK; 2 Emory University School of Medicine, Atlanta, GA USA; 3 Department of Zoology, University of British Columbia, Vancouver, Canada; 4 Department of Microbiology & Immunology, University of British Columbia, Vancouver, Canada

**Keywords:** Symbiosis, Benefits, Environmental stress, Exploitation, Host-symbiont interactions, Microbial regulation

## Abstract

Symbiosis can benefit hosts in numerous ways, but less is known about whether interactions with hosts benefit symbionts—the smaller species in the relationship. To determine the fitness impact of host association on symbionts in likely mutualisms, we conducted a meta-analysis across 91 unique host-symbiont pairings under a range of spatial and temporal contexts. Specifically, we assess the consequences to symbiont fitness when in and out of symbiosis, as well as when the symbiosis is under suboptimal or varying environments and biological conditions (e.g., host age). We find that some intracellular symbionts associated with protists tend to have greater fitness when the symbiosis is under stressful conditions. Symbionts of plants and animals did not exhibit this trend, suggesting that symbionts of multicellular hosts are more robust to perturbations. Symbiont fitness also generally increased with host age. Lastly, we show that symbionts able to proliferate in- and outside host cells exhibit greater fitness than those found exclusively inside or outside cells. The ability to grow in multiple locations may thus help symbionts thrive. We discuss these fitness patterns in light of host-driven factors, whereby hosts exert influence over symbionts to suit their own needs.

**Supplementary Information:**

The online version contains supplementary material available at 10.1007/s13199-024-00984-6.

## Introduction

Long-term associations with symbionts, or symbioses, have had a major influence on the evolution of life on Earth (Margulis and Fester 1991). In beneficial symbioses, symbionts provide hosts with nutrients they would not otherwise be able to utilize (Douglas 1998), with protection from harsh conditions or enemies (Latef et al. 2016; Corbin et al. 2017; King 2019), or with general development and maturation (McFall-Ngai 2002). By contrast, the symbiont is often assumed to benefit from the association, such as provisioning of nutrients and stable environments by the host (Wollenberg and Ruby 2012; Feng et al. 2019)), but recent evidence suggests that associations previously assumed to be mutualistic are not actually beneficial for symbionts (McCutcheon et al. 2019).

From an evolutionary perspective, a mutualism occurs when both partners exhibit a net fitness increase when in symbiosis compared to when free-living. However, symbioses are often context-dependent. Hosts might only benefit under certain ecological contexts (Drew et al. 2021). Similarly, variation in symbiont fitness can be attributed to multiple factors: host biology, symbiont biology, the environment, or some combination thereof (Dossi et al. 2014; López-Madrigal and Duarte 2020). Benefits provided to the symbiont might depend on whether the symbiosis occurs under optimal conditions. For example, hosts may support symbiont growth only when the symbiosis is under perturbation. These conditions include lack of resources, presence of enemies, and co-colonization of multiple symbiont strains, all of which can be stressful for the host (Scarborough et al. 2005; Lau et al. 2012; Oliver et al. 2013; Weldon et al. 2019). In addition to environmental factors, symbiont fitness can also vary over time, including on the scale of a host generation. Symbionts may accumulate across the duration of the symbiosis, or gain more space to grow as hosts develop (Wollenberg and Ruby 2009; Kikuchi et al. 2011). Despite the critical roles symbionts can have in host health and adaptation, it is generally unclear whether symbiosis confers a fitness advantage to symbionts (Douglas and Smith 1989; Garcia and Gerardo 2014).

Here, we test the assumption that symbionts exhibit fitness gains in beneficial symbioses and are robust to perturbation. We examine the effects of environmental and temporal contexts on symbiont fitness in associations where the symbiont provides benefits to the host in at least some contexts. Specifically, our main hypotheses are that symbiont fitness is greater (Hypothesis 1) when in symbiosis as opposed to in a free-living state, and (Hypothesis 2) when the symbiosis is under non-stressful conditions. Alternatively, symbiont fitness could be greater when the symbiosis is under non-optimal conditions. Our third main hypothesis (Hypothesis 3) is that greater symbiont fitness is more common in older hosts. Across time, greater within-host symbiont density is expected as the host has more resources to support symbiont growth (Feng et al. 2019; Fronk and Sachs 2022). An increase in host size over time would also provide more space for symbiont growth (*e.g.,* (Kerwin et al. 2021)).

To test these hypotheses, we conducted a literature search for studies measuring symbiont fitness to evaluate whether and how fitness varies in different environments and throughout host development. We collected data on aspects of host biology, including kingdom (Bermudes and Margulis 1987), reproductive mode (Law and Lewis 1983), and generation time (Takahashi 2016), which may play roles in moderating symbiont fitness. We also collected information on symbiont traits, including symbiont diversity (Foster et al. 2017), location of symbiont on/in hosts (Chomicki et al. 2020), and genome size (Fisher et al. 2017), in addition to the type of association and level of dependence on the host (Fisher et al. 2017), to determine whether symbiont fitness varies for these categories. These variables are summarized in Table 1.

**Table 1:** We examined moderator variables in three analyses that may have an impact on symbiont fitness. Variables include those relating to the host (*e.g.*, kingdom, life stage, reproduction, generation time), the symbiont (*e.g.*, diversity, location, dependence on host, and genome size), or both (environment and type of association). All moderator variables were examined for all three main hypotheses, except host generation time and host life stage, which were done only for H2 and H3, respectively, and dependence on host, which were not done for H1

Moderator	Description
Host kingdom	The taxonomic rank of the host.
Type of association	Function of the symbiont. If unknown, at least commonly found associated with the host without known evidence of harmful effects.
Dependence on host	Whether symbiont depends on host to grow.
Symbiont diversity	Diversity of symbiont species usually found associated with host in nature. From one (*e.g.*, bobtail squid-*Vibrio*), to few (*e.g.*, *Drosophila*), to many (*e.g*., mice).
Host reproduction	Reproductive mode of host.
Location of symbiont	Where symbiont is usually found when associated with host.
Symbiont genome size	Genome size of symbiont. If unknown or species not indicated, the mean of closest relatives sequenced was used.
Host generation time	Generation time of host, in years.
Host life stage	The host life stage(s) under which symbiont fitness was measured.

## Materials and methods

### Literature search and data collection

To evaluate symbiont fitness across different contexts, we conducted a literature search on ISI Web of Science. We used a combination of search terms relating to symbiont fitness, host factors, and host-microbe interactions (specific terms are found in Figure S1). We then identified additional studies by looking through the references of relevant papers and the studies that cited these papers. We included papers that met the following criteria in our analyses:

1. The study measured symbiont fitness in different environments (either outside/without host or under varying abiotic/biotic conditions, such as temperature, resources, or presence of other species) or the study measured symbiont fitness under at least two different time points across the lifespan of the host.
2. The symbiont being examined was considered a beneficial symbiont (e.g., providing the host with tangible or fitness benefits under some context). If its function was unknown, it was at least commonly found in the host population and did not show signs of parasitism.
3. The symbiont was classified at least at the family level as broader classifications did not allow for more specific information to be discerned about the symbiont.
4. Information existed for the variance of each fitness mean estimate.

A plot of all effect sizes from eligible studies, including those removed from the analyses (*i.e.,* those meeting all but the last criterion), is shown in Figure S2.

**Fig. 1:**
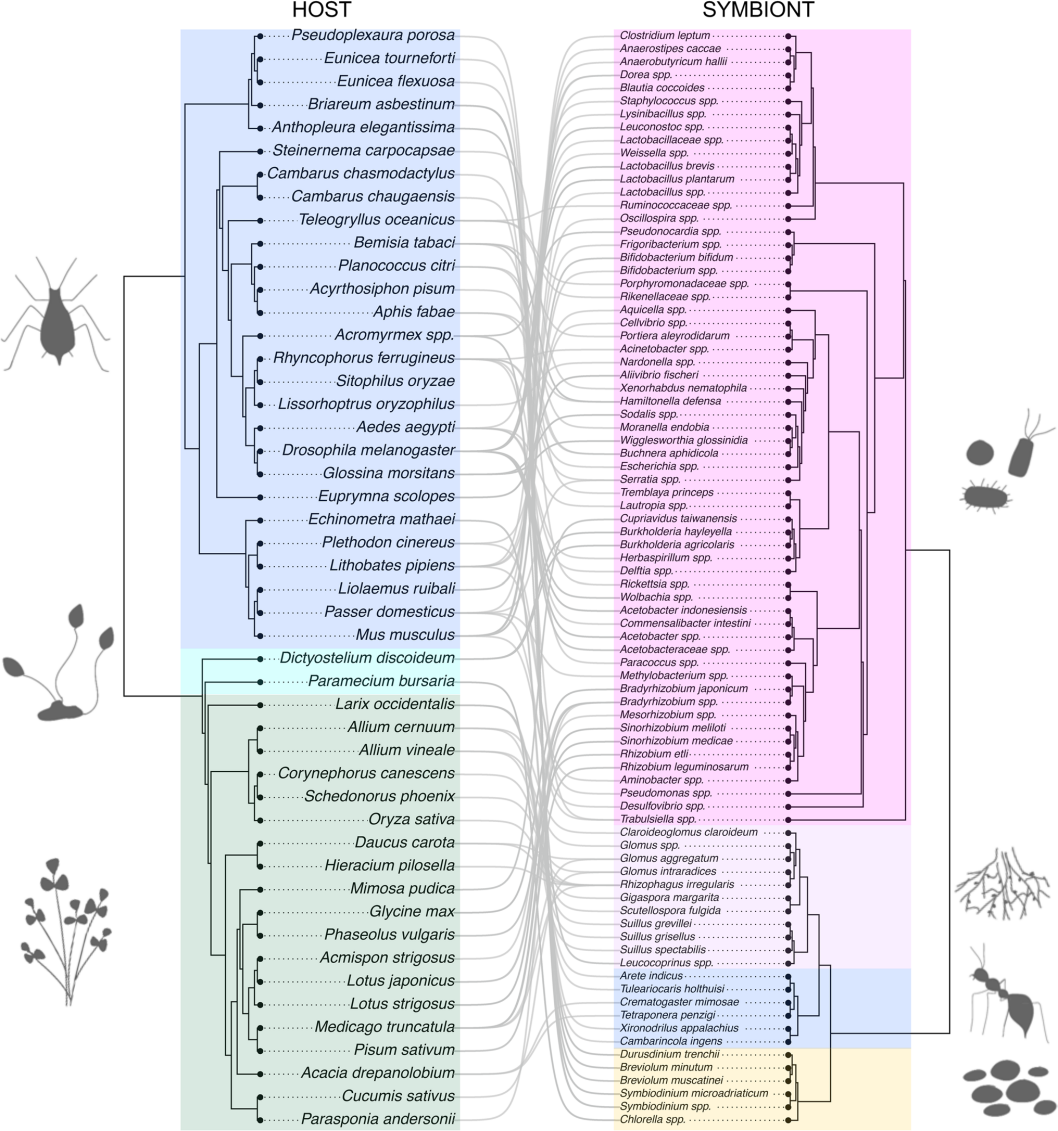
Hosts and symbionts included in meta-analysis. Phylogenetic trees of hosts (left, 47 species) and symbionts (right, 78 species). Colors represent each host or symbiont type (blue = animal, cyan = unicellular eukaryotes, green = plant, pink = bacteria, light purple = fungus, yellow = alga). Gray lines indicate host-symbiont associations (91 unique pairings) examined in included studies

The search resulted in 63 studies (161 effect sizes) that matched our inclusion criteria from studies published between 1994 (the earliest study meeting our criteria) and 2020 (when we started the literature search). For each study, we extracted data using WebDigiPlot (Rohatgi 2021), or contacted authors if relevant information or raw data were not available. We then parsed out the results according to the conditions under which fitness was measured: 11 studies (20 effect sizes) were used in the host association analysis, 50 studies (119 effect sizes) were used in the environment analysis, and 25 studies (42 effect sizes) were used in the time analysis. We also created a subset of the environmental dataset to include only intracellular symbionts (17 studies, 47 effect sizes) to examine how symbiont fitness changes when confined inside host cells. While the majority of the symbionts in our analysis are microbial (e.g., bacteria and fungi), we also included animal symbionts. In such cases, the symbiont is the species that has multiple individuals in symbiosis with one host individual. For example, in the crayfish-branchiobdellidan worm cleaning symbiosis (Thomas et al. 2013), the worm is the symbiont because multiple worms inhabit one crayfish individual. We identified 47 host species and 78 symbiont species from our search, resulting in 91 unique host-symbiont pairings. Host and symbiont phylogenies are included in the meta-analyses (Fig. 1, Table S1), while the types of host and symbiont are in Figures S3A and S3B, respectively.

### Effect size calculation

To test Hypothesis 1 (whether symbiont fitness is greater outside of symbiosis), we collected fitness measurements for symbionts from studies where symbionts are inside or on the surface of the host *vs.* when they are outside of the host (where the host may still be present); or from studies where the host is present *vs.* absent. While the two contexts (inside *vs.* outside and presence *vs.* absence of host) may provide different insights into the role of the host on symbiont fitness (e.g., presence *vs.* absence of host control for the environment and volume in which symbionts grow), there are not enough studies for either context to be analyzed separately. We then calculated the percent change in symbiont fitness (the effect size) using the formula:

$$\%\;change\;in\;symbiont\;fitness= \frac{{(fitness)}_{in\;symbiosis}- {(fitness)}_{out\;of\;symbiosis}}{{(fitness)}_{in\;symbiosis}}$$

To test Hypothesis 2 (whether fitness is greater when the symbiosis is under stress), we collected fitness measurements for the symbiont in a control environment and in an alternative environment. We designated a treatment as the “control” environment when the symbiont interacted with its host at ambient conditions in the absence of other organisms (*i.e.*, those present in the alternative environment); this is representative of the “focal” symbiosis between host and symbiont. The alternative environment indicates one where the symbiosis is experiencing suboptimal or stressful conditions, having negative impacts on host or symbiont performance or fitness (Schulte 2014), or may affect the stability of the interaction (e.g., causing one partner to be lost), relative to the control condition. We used the formula:

$$\%\;change\;in\;symbiont\;fitness=\frac{{(fitness)}_{control}- {(fitness)}_{alternative\;environment}}{{(fitness)}_{control}}$$

To test Hypothesis 3, in that symbiont fitness decreases as hosts age, we considered the “control” treatment to be when the host is younger, using the formula:

$$\%\;change\;in\;symbiont\;fitness=\frac{{(fitness)}_{younger\;host}- {(fitness)}_{older\;host}}{{fitness)}_{younger\;host}}$$

Our study included a diverse range of fitness measures, including area of host colonized, colony forming units (CFU), count, density, fluorescence, growth rate, nodule number, sequences and survival. To compare effect sizes, we converted the difference in fitness means between the control and experimental treatments from each study into a percentage for our analyses (similar to (Fisher et al. 2017)) to standardize across the different fitness measurements (Figure S3C), which vary in scale across systems (*e.g*., nodule number *vs.* number of colony forming units). To calculate the variance of the percent change, we used the formula from Appendix 6 of Haney et al. (2007). Lastly, we cube-root transformed (to preserve positive and negative values) the final percent change and variance values due to the presence of several very large effect sizes.

### Symbiont and host phylogeny construction

Phylogenetic relatedness can be a source of non-independence between effect sizes—closely related symbionts may respond in the same way to selective pressures, or closely related hosts can similarly affect symbionts (Murfin et al. 2015). To account for phylogenetic non-independence in our models, we constructed phylogenies of the symbionts and hosts included in our analyses. We pruned the tree available at the Open Tree of Life (OTL) with the R packages *rotl* and *ape* to build trees containing our species of interest and visualized them using the *phytools* package (Revell 2012; Michonneau et al. 2016; Paradis and Schliep 2019). When a species was not found in OTL, we found the closest relative available in the genus or family (10 instances; Table S2), then substituted it in place of the missing species. Because some species have more than one effect size, we generated trees such that each species was classified at the population level to match with their corresponding effect size. The phylogenetic distances between “populations” of the same species were <  < 0.00001 (effectively zero), but the population-level designation allow us to distinguish between populations from different studies. We then converted the phylogenies into correlation matrices assuming Brownian motion to incorporate into our phylogenetically-informed meta-analyses.

### Statistical analysis

We conducted separate analyses for the host association, environment, and time datasets using the R package *metafor* (Viechtbauer 2010)*.* We built multi-level mixed-effects models using the rma.mv function restricted maximum likelihood estimation of parameters. To account for some studies having multiple effect sizes, we included between-study effects and within-study effects as random factors (Noble et al. 2017). We ran the models treating symbiont phylogeny as a random effect, then re-ran the models treating host phylogeny as a random effect. Because results were qualitatively the same for both phylogenies, we present the results for incorporation of symbiont phylogeny. We then conducted moderator analyses using a Wald-type test (QM statistic) to examine the effects of specific variables on symbiont fitness (Table 1) (Viechtbauer 2010). For the environment dataset, we also examined a subset of effect sizes that belonged to intracellular symbionts and included host generation time as a moderator variable. For hosts associated with multiple symbionts (or vice versa), we ran the analysis for each unique host-symbiont pairing or for each unique symbiont. For symbionts with multiple effect sizes, we used the mean effect size. To identify potential outliers, we calculated Cook’s distance (D) for each analysis and removed effect sizes that had D greater than three times the mean (Cook 1977). All statistical analyses were conducted in R (R Core Team 2021).

## Results

### Host association has no significant effects on symbiont fitness

**Fig. 2:**
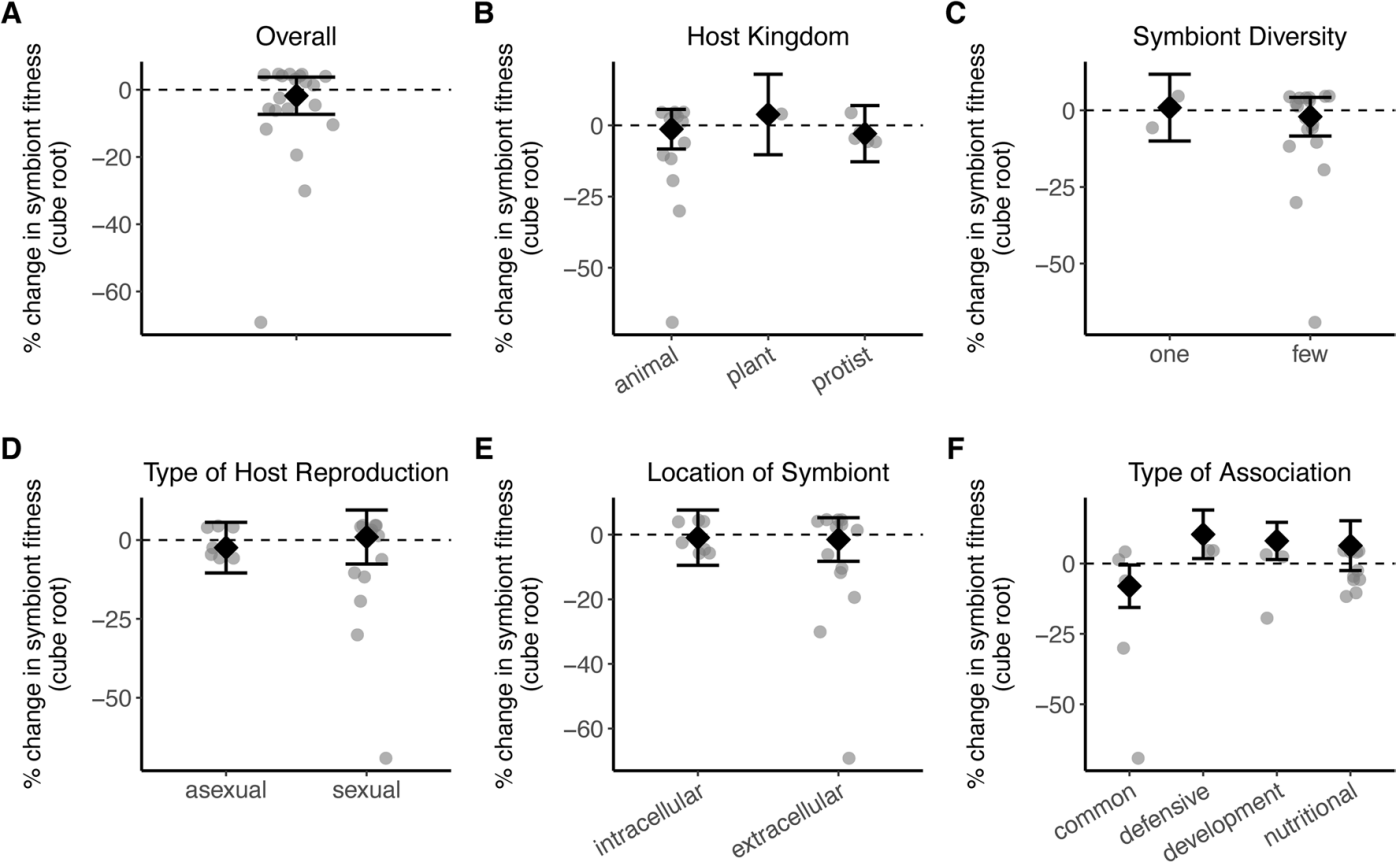
The effect of host association on percent change in symbiont fitness. A) The overall effect of being in symbiosis on symbiont fitness. The percent change in symbiont fitness when host-associated across different B) host kingdoms, C) symbiont diversity level, D) host reproductive modes, E) sites of colonization, and F) types of association. Each data point represents an effect size (n = 20 effect sizes); those below the dashed line indicate fitness being greater when out of symbiosis. Error bars indicate 95% confidence intervals

The results did not support our first hypothesis: there was no significant effect of host association on symbiont fitness (z = -0.627, p = 0.531; Fig. 2A). Neither the direction or magnitude of the effect size was influenced by host kingdom (QM = 0.755, df = 2, p = 0.686; Fig. 2B), symbiont diversity (QM = 0.027, df = 1, p = 0.871; Fig. 2C), host reproductive mode (QM = 0.046, df = 1, p = 0.831; Fig. 2D), symbiont location (QM = 0.046, df = 1, p = 0.831; Fig. 2E), or genome size (QM = 0.407, df = 1, p = 0.524). The type of association and symbiont dependence on host were marginally significant (QM = 6.809, df = 3, p = 0.078 and QM = 3.211, df = 1, p = 0.073, respectively; Figs. 2F and 2G). Table S3 contains the results for all overall and moderator analyses, with all effect sizes or with outliers removed.

### Animal-associated symbionts have opposing trends to protist-associated symbionts

**Fig. 3:**
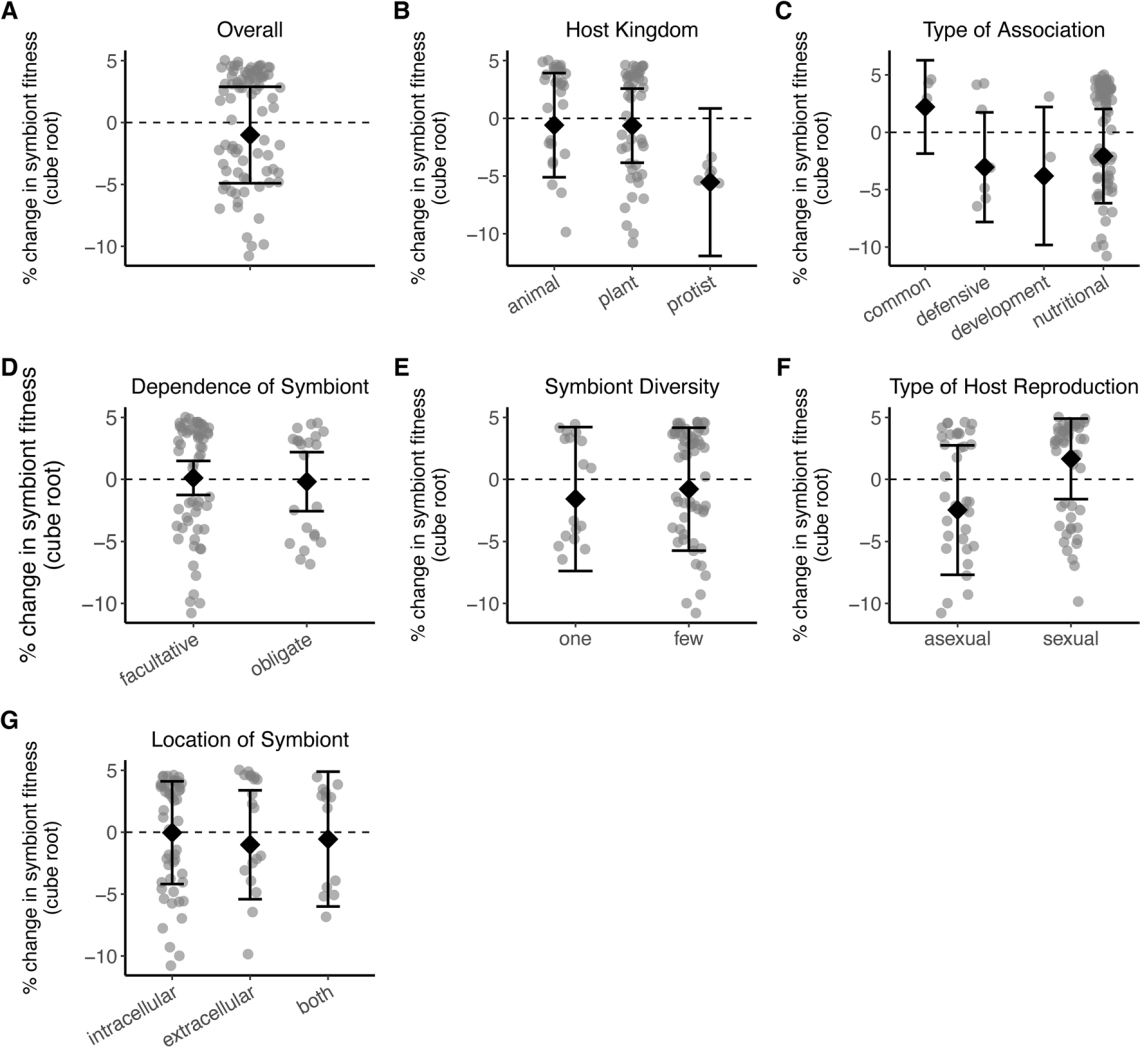
The effect of environment on percent change in symbiont fitness. A) The overall effect of alternative environments, where the symbiosis experienced suboptimal or stressful conditions, on symbiont fitness. The percent change in symbiont fitness in different environments across B) host kingdoms, C) types of association, D) degrees of symbiont dependence on host, E) levels of symbiont diversity, F) host reproductive modes, and G) sites of colonization. Each data point represents an effect size (n = 99 effect sizes); those below the dashed line indicate fitness being greater in the alternative environment. Error bars indicate 95% confidence intervals

**Fig. 4:**
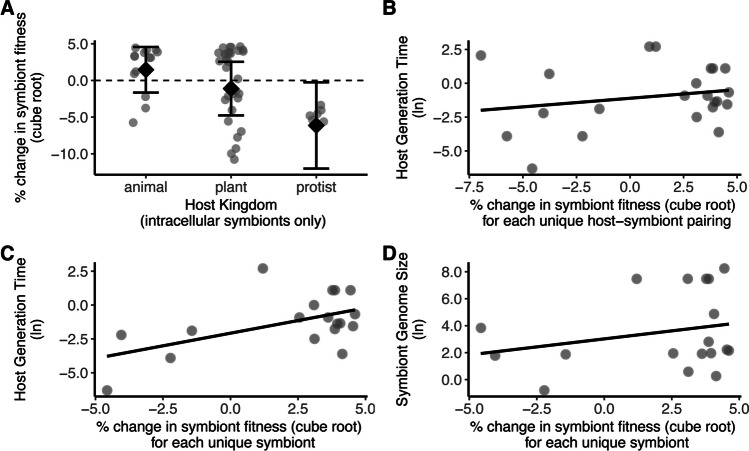
The effect of environment on percent change in intracellular symbiont fitness. A) The percent change in symbiont fitness across host kingdoms. Each data point represents an effect size (n = 51 effect sizes); those below the dashed line indicate fitness being greater in the alternative environment. Error bars indicate 95% confidence intervals. B) Correlation between host generation time and symbiont fitness for unique host-symbiont pairings. C) Correlation between host generation time and symbiont fitness for each unique symbiont. D) Correlation between symbiont genome size and symbiont fitness for each unique symbiont

There was no significant overall effect of environment on symbiont fitness (Hypothesis 2, z = -0.509, p = 0.611; Fig. 3A). The direction and magnitude of the effect size were not influenced by host kingdom (QM = 2.899, df = 2, p = 0.235; Fig. 3B), type of association (QM = 2.124, df = 3, p = 0.547; Fig. 3C), symbiont dependence on host (QM = 0.025, df = 1, p = 0.874; Fig. 3D), symbiont diversity (QM = 0.358, df = 2, p = 0.836; Fig. 3E), host reproductive mode (QM = 0.985, df = 1, p = 0.321; Fig. 3F), location of symbiont (QM = 0.402, df = 2, p = 0.818; Fig. 3G), or genome size (QM = 0.641, df = 1, p = 0.423). However, when outliers were removed, host kingdom was significant (QM = 7.300, df = 2, p = 0.026), particularly for intracellular symbionts (QM = 12.25, df = 2, p = 0.002; Fig. 4A). Protists harbored symbionts that performed better when in suboptimal environments. Symbiont fitness may be tied to host cell division for intracellular symbionts. For example, unicellular hosts and their symbionts undergo synchronized cell division (Kadono et al. 2004; Motta et al. 2010), where a shorter host generation time may prevent symbionts from accumulating. We therefore examined whether host generation time was correlated with change in symbiont fitness and found no correlation when accounting for each unique host-symbiont pairing (QM = 2.191, p = 0.139; Fig. 4B). However, when accounting for each unique symbiont (regardless of the host associated with the symbiont), symbiont fitness was positively correlated with host generation time (QM = 5.036, p = 0.025; Fig. 4C) and symbiont genome size (QM = 4.524, p = 0.033; Fig. 4D).

### Symbiont fitness is greater in older hosts

**Fig. 5:**
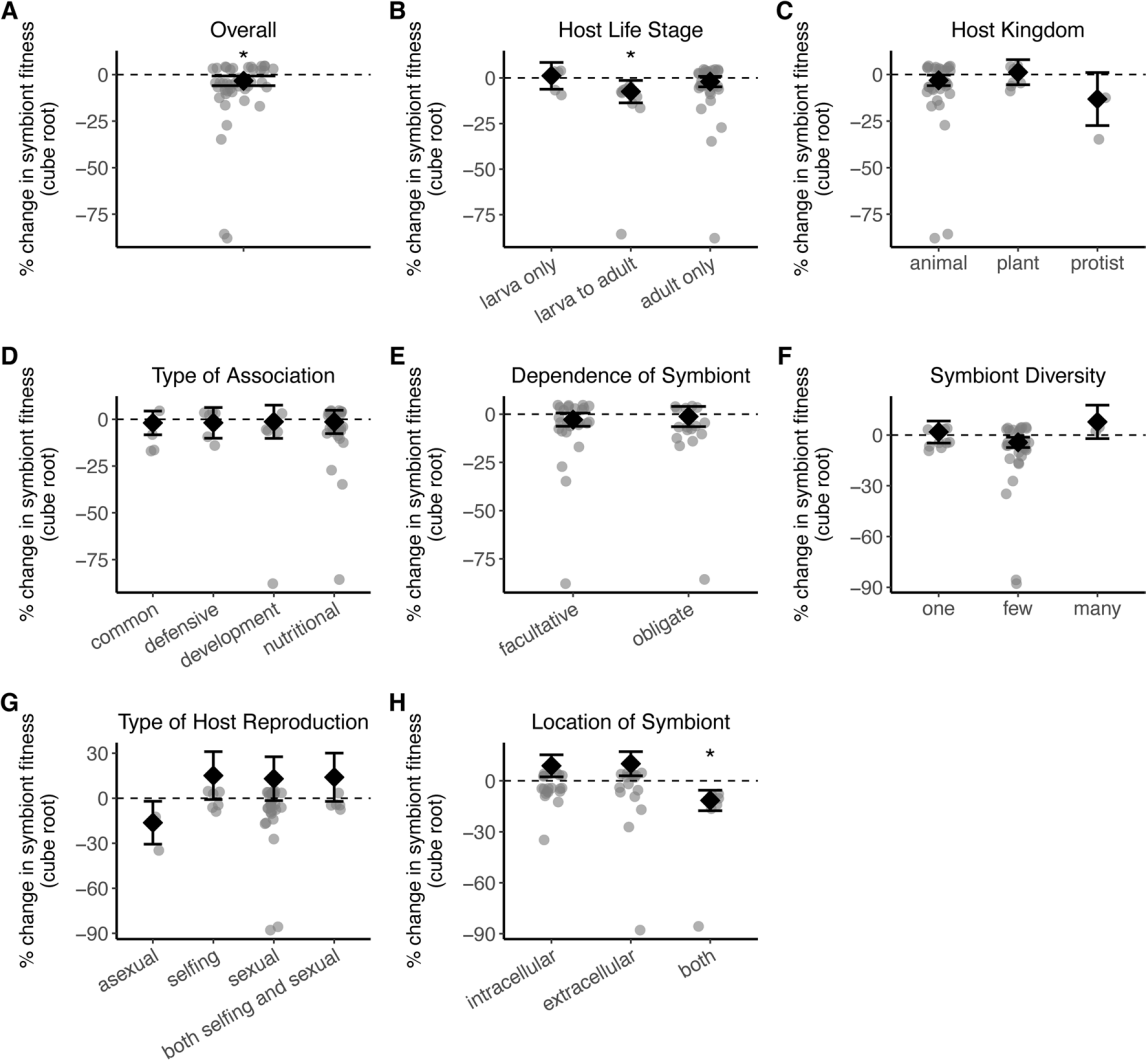
The effect of time on percent change in symbiont fitness. A) The overall effect of time on symbiont fitness. The percent change in symbiont fitness across host development among B) host life stages, C) host kingdoms, D) types of association, E) degrees of symbiont dependence on host, F) symbiont diversity levels, G) host reproductive types, and H) sites of colonization. Each data point represents an effect size (n = 42 effect sizes); those below the dashed line indicate fitness being greater when hosts are older. Error bars indicate 95% confidence intervals. *p < 0.05

To test Hypothesis 3, we examined how symbiont fitness changes over time. As hosts develop and increase in size, symbionts may acquire more space to grow or more time to proliferate (Wollenberg and Ruby 2009; Kikuchi et al. 2011). Our results supported this hypothesis: there was a significant effect of time on the percent change in symbiont fitness, where fitness increased overall in older hosts (z = -2.524, p = 0.012; Fig. 5A). However, there were some studies in which symbiont fitness increased to a certain point, then subsequently declined (Rio et al. 2006; Hamidou Soumana et al. 2013; Vigneron et al. 2014; Zhao et al. 2018; Garcia et al. 2019). Since our criterion was to record symbiont fitness at the timepoint when hosts were the oldest in each study, the effect sizes from the above studies could have been greater if peak fitness had been recorded instead. Host life stage was a significant moderator variable (QM = 6.235; df = 2, p = 0.044; Fig. 5B), with symbiont fitness tend to be greater in adults when compared to the juvenile stage. Conversely, host kingdom (QM = 3.539, df = 2, p = 0.170; Fig. 5C), type of association (QM = 0.235, df = 3, p = 0.972; Fig. 5D), symbiont dependence on host (QM = 0.213, df = 1, p = 0.645; Fig. 5E), symbiont diversity (QM = 2.467, df = 2, p = 0.291; Fig. 5F), host reproductive mode (QM = 3.569, df = 3, p = 0.312; Fig. 5G), and symbiont genome size (QM = 0.006, df = 1, p = 0.940) did not influence the direction or magnitude of the effect size.

Symbionts able to grow outside host cells (which include the space between cells within a host or on host surfaces) should perform better than those confined to host cells. This hypothesis was partially supported: symbionts found both inside and outside cells tended to have greater fitness when hosts were older, whereas such pattern was not found for exclusively intracellular or extracellular symbionts (QM = 8.609, df = 2, p = 0.014; Fig. 5H). Similar to the host association and environmental analyses, there was high variation in terms of symbiont fitness across studies and systems.

### Outlier sensitivity analysis

Removal of outliers did not change the mean significance level for all but two of our results (Table S3), where the host kingdom moderator influenced symbiont fitness in different environments, and this effect was greater in intracellular symbionts.

## Discussion

Overall, we did not find support for the hypothesis that symbionts gain a fitness benefit when host-associated. This finding could be due to ongoing conflicts between hosts and symbionts. Indeed, mutualism has been viewed as exploitation between partners that result in a net fitness benefit for both (Herre et al. 1999). Exploitation by symbionts is predicted due to their rapid evolution, similarities to pathogens, and the context-dependency under which benefits are provided to hosts (Davitt et al. 2011; Sachs et al. 2011; Weldon et al. 2013). Conversely, research has also indicated that hosts can take advantage of their symbionts before reciprocating benefits (Sorensen et al. 2019). Hosts can modify the growth of their symbionts in a variety of ways (Box 1)—from farming (Currie 2001), to compartmentalization (Chomicki et al. 2020), to expulsion (Thomas et al. 2013), to active culling (Vigneron et al. 2014; Piquet et al. 2019), increasing or decreasing symbiont abundance to suit host interests. Consequently, such regulation of symbiont population size may have resulted in no net fitness gain or loss across studies included in our analyses.

**BOX 1:** Host regulation of symbiont reproduction

*Why is having control over the symbiont beneficial for hosts?*
Hosts are likely under selection to regulate symbiont densities (Drew and King 2022; Whittle et al. 2023). By manipulating symbiont reproduction, hosts can procure the symbiont products they require, even at the cost of symbiont survival. Host association can also prevent symbionts from reaching high density and drastically reduce population size compared to when symbionts are free-living, mitigating potential costs of harboring symbionts (Ankrah et al. 2018). For hosts with a complex microbiome, keeping their microbes ‘on a leash’ is critical (Foster et al. 2017). Microbial evolution driven by competition between species must be regulated to foster a beneficial community (Foster et al. 2017; Drew et al. 2021).
*Mechanisms of regulation*
Hosts employ a diverse suite of mechanisms to regulate their symbiont population. In well-established symbioses, hosts have evolved physical structures to confine their symbionts to certain tissues (Chomicki et al. 2020). Indeed, symbionts housed inside host cells are considered some of the most close-knit associations, but residing inside host cells also allows the host more control over its symbiont. For example, hosts can produce chemicals to limit symbiont mobility and cell division and to control the influx of metabolites that the symbiont receives (Wooldridge 2010; Russell et al. 2014). For single-celled hosts (e.g., protists), the replication rate of symbionts can depend on host generation time. In order for symbionts to reproduce, they must synchronize with host cell division, even if it means a reduction in replication rate (Takahashi 2016). Finally, symbionts can be eliminated through active killing by the immune system or apoptosis, expulsion into the external environment, or bottlenecks from parent to offspring transmission (Frank 1996; Baghdasarian and Muscatine 2000; Vigneron et al. 2014; Laurich et al. 2018; Gerardo et al. 2020). Host control mechanisms thus allow hosts to exploit their symbionts to derive maximal benefits.
*Examples*
Corals capture dinoflagellates and through host controlled processes, “farm” the algae in order to obtain their photosynthetic products, incurring a fitness cost to the symbiont in the process (Wooldridge 2010). Indeed, several symbioses involve symbiont farming, where hosts facilitate symbiont growth to establish a nutritional reserve (Hoang et al. 2019). In some legume species, the nitrogen fixing form of rhizobia, bacteroids, are terminally differentiated, where they can no longer reproduce. This loss of replication ability is induced by host factors, which in turn benefits the host in several different ways (Kereszt et al. 2011; Oono et al. 2011). Even if these bacteria can escape into the environment when the host senesces, they are at an evolutionary dead end. Alternatively, in some cnidarian species, hosts preferentially expel dividing algal cells back into seawater, presumably as a way for the host to control its internal algal density (Baghdasarian and Muscatine 2000). In the most extreme case, hosts can steal chloroplasts from their algal symbionts, allowing hosts to perform photosynthesis themselves. This phenomenon, called kleptoplasty, has been found in protists such as ciliates (Hansen et al. 2012), dinoflagellates (Nishitani et al. 2012), and foraminifera (Bernhard and Bowser 1999; Pillet and Pawlowski 2013), as well as in sacoglossan molluscs (Rumpho et al. 2011). These interactions effectively kill the symbiont while they are in symbiosis.

Symbionts not gaining from being in symbiosis may also be partly attributed to how fitness is quantified across studies. Measuring symbiont fitness inside versus outside the host is one way in which benefits are evaluated, but the space occupied by the symbiont is vastly different in the host and external environment (Douglas and Smith 1989). For example, there is less space for growth inside the host. Comparisons would have to be made in an environment of comparable volume to the host while also taking into consideration symbiont density in these spaces. The tested environment may also not be representative of the conditions in which symbionts are found in nature (*e.g*., rich media). Recent efforts have developed methods to compare symbiont populations in environments where the host is either present or absent (Burghardt et al. 2018; Burghardt 2019; Garcia et al. 2019; Mendoza-Suárez et al. 2020). With this approach, the volume of space is the same across all measurements and both partners occupy the same type of environment. However, there is a lack of studies measuring symbiont fitness inside *vs.* outside the host and in the presence *vs.* absence of hosts in general.

Our literature search yielded few studies where symbiont fitness was measured in and out of symbiosis, regardless of whether they were outside of the host or growing in the absence of the host. The lack of these studies could be representative of the proportion of symbionts able to thrive without host help. Because these symbioses lacked quantitative information on symbiont fitness, we were unable to include them. Well-known examples of these associations from the literature (*e.g*., (Fisher et al. 2017)) are presented in Figure S6, consisting of 60 unique host species and 35 unique symbiont species. Conversely, the lack of studies may be due to less emphasis on symbiont traits in the literature. There is a need for more experiments investigating the role of host association on symbiont fitness in general.

### Symbiont fitness across environments

We found that single-celled hosts tend to harbor symbionts that incur costs under benign conditions compared to when stressed. The difference in symbiont abundance could be due to attributes of single-celled and multicellular hosts themselves. For example, unicellular eukaryotes (*i.e.*, protists) are considered extant models for when symbioses first evolved (Gavelis and Gile 2018). As such, unicellular hosts may lack the ability to regulate their symbiont populations, thus more symbionts are free to grow when conditions are unstable. Conversely, animal and plant hosts potentially have had a longer evolutionary history with their symbionts and have evolved more robust methods of regulation (e.g., facilitation through nutrient provisioning (Feng et al. 2019) and specialized cells for housing symbionts (McFall-Ngai 2008; Chomicki et al. 2020)). These mechanisms allow for high symbiont population sizes when conditions are optimal, and low population sizes when conditions are stressful.

We also found that longer host generation time is correlated with higher intracellular symbiont fitness when under optimal conditions. Protists generally have shorter generation times than animals and plants. As host age is an important factor in symbiont fitness, the rapid turnover rates and shorter lifespan of protist hosts compared to animal hosts may not allow sufficient time for symbionts to accumulate in vivo. Alternatively, symbiont growth may not be host-driven. Symbionts may limit their own cell division within hosts when both partners benefit (Uchiumi et al. 2019). Having a larger genome tended to benefit symbionts in optimal conditions, which could also serve to reduce their dependence on the host for growth (Fisher et al. 2017). Regardless of the underlying mechanism, protist-microbe symbioses appear to support the stress gradient hypothesis. This hypothesis predicts that positive interactions between the partners will increase as conditions become more stressful (Bertness and Callaway 1994; Maestre et al. 2009; O’brien et al. 2018; Adams et al. 2022), where high levels of stress favors increased benefits for both partners. These findings may however be due to study limitations; single-celled hosts are more amenable to experiments and are model systems used in most studies of host exploitation (e.g., Lowe et al. 2016; Sorensen et al. 2019)).

### Symbiont fitness across time

Several studies in our analysis saw symbiont fitness at a maximum before declining (Rio et al. 2006; Hamidou Soumana et al. 2013; Vigneron et al. 2014; Zhao et al. 2018; Garcia et al. 2019). It is possible that the hosts in these studies can regulate symbiont populations. One such mechanism is through the immune system, which is dynamic with host age. As hosts mature, the immune system may become more developed and robust to proliferating microbes (*i.e.,* symbionts and pathogens) (Davidson et al. 2004; Johnston and Rolff 2015). Indeed, the largest differences in symbiont fitness were found between juvenile and adult life stages in our meta-analysis, with adults typically harboring more. As hosts age and may no longer require the symbiont, reducing symbiont population size could decrease costs associated with high symbiont titers (Vigneron et al. 2014; Chong and Moran 2016). Furthermore, symbionts found inside and outside host cells increased in abundance in older hosts—the ability to proliferate in multiple locations may allow symbionts to escape host regulation mechanisms. However, the same immune system is weakened as hosts senesce. This process may allow for accumulation of symbionts toward a level that is detrimental for hosts (Portal-Celhay et al. 2012). Identifying further links between host immunity, ageing, and microbial growth would advance understanding of temporal dynamics of symbiont density and the impact on host health.

### Challenges in assessing symbiont fitness

Many effect sizes were on the order of magnitudes greater than the mean. The high variation across studies may be due to conceptual challenges of ascertaining symbiont fitness. Whether the symbiont benefits may depend on the context under which the symbiosis is scrutinized. At the molecular or physiological scale, provisions of host metabolites might suffice as a beneficial mechanism without a need for fitness measurements. However, from an ecological or evolutionary standpoint, increased fitness in symbiosis *versus* outside of symbiosis may be a better indicator of a reciprocal mutualism (Law and Dieckmann 1998; Mushegian and Ebert 2015). A classical test of mutualism calls for measuring fitness of one partner in the absence of the other (Douglas and Smith 1989; Wooldridge 2010; Mushegian and Ebert 2015), but this is likely not possible for symbionts that cannot be grown without hosts. Furthermore, any benefits identified from being in symbiosis for these symbionts may be confounded by their dependence on the host, and dependence may not actually require the exchange of benefits (Douglas and Smith 1989; Douglas 2015).

Whether the symbiont benefits from host association becomes even more complicated once a microbial community is involved. Symbiont fitness may not solely depend on the host but also other microbes present (Chamberlain et al. 2014; Mushegian and Ebert 2015; Song et al. 2020). Microbes can act as in vivo competitors or facilitators in a community context, further complicating the assessment of the fitness of any one particular symbiont. Moreover, symbiont persistence in host populations may not rely on total abundance in any individual host as much as transmission between hosts, whether from parent to offspring or dispersal to new environments (Lee and Ruby 1994; Brock et al. 2011; Ebert 2013; Thutupalli et al. 2017). Finally, the same fitness proxy cannot be applied across all symbiont types (e.g., percent of host cells occupied by the symbiont *vs.* nodule count), as with most multicellular hosts (e.g., survival or fecundity (Fisher et al. 2017)). Taken together, these perspectives suggest that there may not be a consensus as to how fitness should be evaluated for all symbionts.

## Conclusion

Mutualistic symbiosis is often thought to involve reciprocal benefits. However, the mechanism underlying the interaction can be antagonistic—symbionts may not be free to proliferate while host-associated. Mechanisms of host regulation remain to be explored in many systems. Determining whether a symbiont is gaining from the association may require a combination of approaches to tackle. Extending research to additional and less-studied taxa will inform a better understanding of forces shaping symbiont fitness in general. Lastly, focusing on symbionts in and out of relationships with their multicellular hosts will lend further insight into the processes governing symbiont growth, such as elements of the host that change during senescence.

### Supplementary Information

Below is the link to the electronic supplementary material.

Supplementary file1 Figure S1. PRISMA flowchart of steps taken to identify studies to be used in host association, environmental, and time analyses. Figure S2. All effect sizes from eligible studies. Figure S3. Hosts, symbionts, and type of data included in analyses. Figure S4. Phylogenies of unculturable symbionts and their hosts. Table S1. Hosts and symbionts included in meta-analysis. Table S2. Closest relative to symbiont species included in meta-analysis. Table S3. Results of overall and moderator analyses, with all effect sizes or with outliers removed. (DOCX 1217 KB)

## Data Availability

Data used for analyses is publicly available in Mendeley Data: 10.17632/4rj49vkgtd.1.
