# Supplementary material for: The impacts of host association and perturbation on symbiont fitness
Source: Symbiosis. 2024 Apr 2;92(3):439–51. doi: 10.1007/s13199-024-00984-6 (PMC11039428; doi:10.1007/s13199-024-00984-6)
Supplement: Supplementary file 1 — Supplementary file1 Figure S1. PRISMA flowchart of steps taken to identify studies to be used in host association, environmental, and time analyses. Figure S2. All effect sizes from eligible studies. Figure S3. Hosts, symbionts, and type of data included in analyses. Figure S4. Phylogenies of unculturable symbionts and their hosts. Table S1. Hosts and symbionts included in meta-analysis. Table S2. Closest relative to symbiont species included in meta-analysis. Table S3. Results of overall and moderator analyses, with all effect sizes or with outliers removed. (DOCX 1217 KB) [file 13199_2024_984_MOESM1_ESM.docx]

**SUPPLEMENTAL FIGURES**

**Figure S1.** PRISMA flowchart of steps taken to identify studies to be used in host association, environmental, and time analyses. Data unsuitable indicates the study did not meet one or more inclusion criteria described in the Materials and Methods.


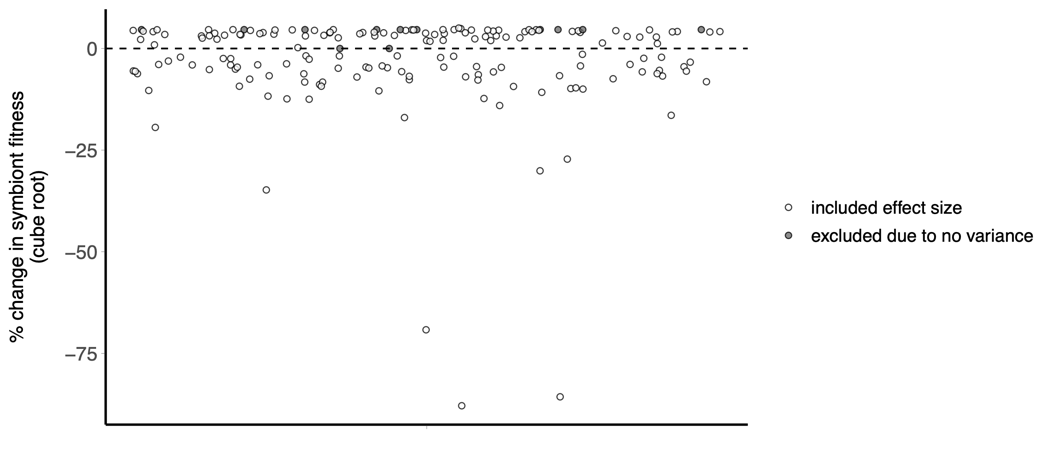


**Figure S2**. All effect sizes from eligible studies: those that met all inclusion criteria (open circles) or removed from analyses due to lack of variance information (filled circles).

**Figure S3.** Hosts, symbionts, and type of data included in analyses. Counts of a) host and b) symbiont types, respectively. c) Types of symbiont fitness proxies used in the studies meeting our inclusion criteria (area colonized refers to percent of host tissue colonized by symbiont, CFU refers to colony forming units, nodule number and volume refer to those formed by rhizobia on legumes, sequences refer to quantification of symbiont DNA through methods such as 16S sequencing or qPCR). Y-axis indicates the number of times each fitness proxy is used for an effect size.


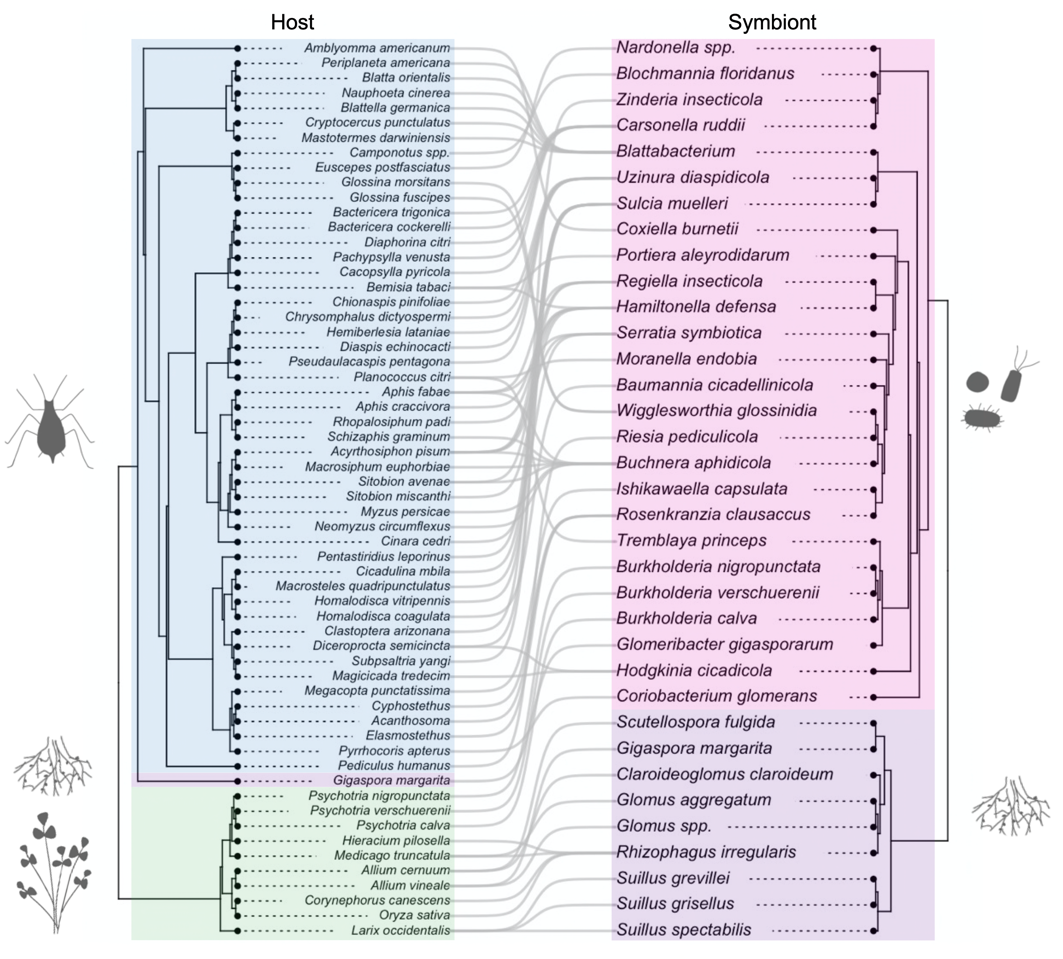


**Figure S4.** We identified well-known examples of unculturable symbionts and built phylogenies of these symbionts (right) and their associated hosts (left) due to the lack of quantitative data on the fitness of these symbionts outside of symbiosis. Colors represent each host or symbiont type (blue = animal, purple = fungus, green = plant, pink = bacteria). Gray lines indicate associations found in nature.

**SUPPLEMENTAL TABLES**

**Table S1.** Hosts and symbionts included in meta-analysis.

| **Host species** | **Host type** | **Symbiont species** |
| --- | --- | --- |
| Animal hosts | | |
| *Lithobates pipiens* | amphibian | *Aminobacter spp.* |
| *Lithobates pipiens* | amphibian | *Dorea spp.* |
| *Lithobates pipiens* | amphibian | *Aquicella spp.* |
| *Lithobates pipiens* | amphibian | *Frigoribacterium spp.* |
| *Plethodon cinereus* | amphibian | *Pseudomonas spp.* |
| *Plethodon cinereus* | amphibian | *Cellvibrio spp.* |
| *Passer domesticus* | bird | *Methylobacterium spp.* |
| *Passer domesticus* | bird | *Serratia spp.* |
| *Passer domesticus* | bird | *Trabulsiella spp.* |
| *Passer domesticus* | bird | *Dorea spp.* |
| *Euprymna scolopes* | cephalopod | *Aliivibrio fischeri* |
| *Briareum asbestinum* | cnidarian | *Durusdinium trenchii* |
| *Briareum asbestinum* | cnidarian | *Breviolum minutum* |
| *Briareum asbestinum* | cnidarian | *Symbiodinium microadriaticum* |
| *Anthopleura elegantissima* | cnidarian | *Breviolum muscatinei* |
| *Eunicea tourneforti* | cnidarian | *Symbiodinium spp.* |
| *Eunicea flexuosa* | cnidarian | *Symbiodinium spp.* |
| *Pseudoplexaura porosa* | cnidarian | *Symbiodinium spp.* |
| *Cambarus chaugaensis* | crustacean | *Xironodrilus appalachius* |
| *Cambarus chasmodactylus* | crustacean | *Cambarincola ingens* |
| *Echinometra mathaei* | echinoderm | *Arete indicus* |
| *Echinometra mathaei* | echinoderm | *Tuleariocaris holthuisi* |
| *Acyrthosiphon pisum* | insect | *Buchnera aphidicola* |
| *Bemisia tabaci* | insect | *Hamiltonella defensa* |
| *Bemisia tabaci* | insect | *Portiera aleyrodidarum* |
| *Bemisia tabaci* | insect | *Rickettsia spp.* |
| *Drosophila melanogaster* | insect | *Acetobacter indonesiensis* |
| *Drosophila melanogaster* | insect | *Lactobacillus plantarum* |
| *Drosophila melanogaster* | insect | *Lactobacillaceae spp.* |
| *Drosophila melanogaster* | insect | *Lactobacillus brevis* |
| *Drosophila melanogaster* | insect | *Acetobacteraceae spp.* |
| *Glossina morsitans* | insect | *Sodalis spp.* |
| *Glossina morsitans* | insect | *Wigglesworthia glossinidia* |
| *Lissorhoptrus oryzophilus* | insect | *Nardonella spp.* |
| *Sitophilus oryzae* | insect | *ÔªøSodalis pierantonius* |
| *Aedes aegypti* | insect | *Herbaspirillum spp.* |
| *Aedes aegypti* | insect | *Delftia spp.* |
| *Aedes aegypti* | insect | *Lautropia spp.* |
| *Aedes aegypti* | insect | *Staphylococcus spp.* |
| *Drosophila melanogaster* | insect | *Acetobacter spp.* |
| *Drosophila melanogaster* | insect | *Leuconostoc spp.* |
| *Drosophila melanogaster* | insect | *Wolbachia spp.* |
| *Rhyncophorus ferrugineus* | insect | *Paracoccus spp.* |
| *Rhyncophorus ferrugineus* | insect | *Acinetobacter spp.* |
| *Rhyncophorus ferrugineus* | insect | *Serratia spp.* |
| *Rhyncophorus ferrugineus* | insect | *Trabulsiella spp.* |
| *Rhyncophorus ferrugineus* | insect | *Escherichia spp.* |
| *Teleogryllus oceanicus* | insect | *Ruminococcaceae spp.* |
| *Teleogryllus oceanicus* | insect | *Rikenellaceae spp.* |
| *Teleogryllus oceanicus* | insect | *Porphyromonadaceae spp.* |
| *Drosophila melanogaster* | insect | *Commensalibacter intestini* |
| *Drosophila melanogaster* | insect | *Lysinibacillus spp.* |
| *Drosophila melanogaster* | insect | *Weissella spp.* |
| *Acromyrmex spp.* | insect | *Leucocoprinus spp.* |
| *Acromyrmex spp.* | insect | *Pseudonocardia spp.* |
| *Planococcus citri* | insect | *Moranella endobia* |
| *Planococcus citri* | insect | *Tremblaya princeps* |
| *Aphis fabae* | insect | *Buchnera aphidicola* |
| *Aphis fabae* | insect | *Hamiltonella defensa* |
| *Mus musculus* | mammal | *Bifidobacterium bifidum* |
| *Mus musculus* | mammal | *Blautia coccoides* |
| *Mus musculus* | mammal | *Anaerobutyricum hallii* |
| *Mus musculus* | mammal | *Anaerostipes caccae* |
| *Mus musculus* | mammal | *Desulfovibrio spp.* |
| *Mus musculus* | mammal | *Clostridium leptum* |
| *Mus musculus* | mammal | *Bifidobacterium spp.* |
| *Steinernema carpocapsae* | nematode | *Xenorhabdus nematophila* |
| *Liolaemus ruibali* | reptile | *Oscillospira spp.* |
| *Liolaemus ruibali* | reptile | *Lactobacillus spp.* |
| Plant hosts | | |
| *Allium cernuum* | Allium | *Claroideoglomus claroideum* |
| *Allium cernuum* | Allium | *Scutellospora fulgida* |
| *Allium vineale* | Allium | *Glomus spp.* |
| *Allium vineale* | Allium | *Gigaspora margarita* |
| *Daucus carota* | carrot | *Rhizophagus irregularis* |
| *Daucus carota* | carrot | *Glomus aggregatum* |
| *Cucumis sativus* | cucurbit | *Glomus intraradices* |
| *Hieracium pilosella* | daisy | *Rhizophagus irregularis* |
| *Corynephorus canescens* | grass | *Rhizophagus irregularis* |
| *Oryza sativa* | grass | *Rhizophagus irregularis* |
| *Schedonorus phoenix* | grass | *Glomus intraradices* |
| *Glycine max* | legume | *Bradyrhizobium japonicum* |
| *Pisum sativum* | legume | *Rhizobium leguminosarum* |
| *Lotus japonicus* | legume | *Mesorhizobium spp.* |
| *Lotus strigosus* | legume | *Bradyrhizobium spp.* |
| *Medicago truncatula* | legume | *Sinorhizobium meliloti* |
| *Medicago truncatula* | legume | *Sinorhizobium medicae* |
| *Medicago truncatula* | legume | *Glomus aggregatum* |
| *Medicago truncatula* | legume | *Rhizophagus irregularis* |
| *Acmispon strigosus* | legume | *Bradyrhizobium spp.* |
| *Lotus japonicus* | legume | *Bradyrhizobium spp.* |
| *Phaseolus vulgaris* | legume | *Rhizobium etli* |
| *Mimosa pudica* | legume | *Cupriavidus taiwanensis* |
| *Acacia drepanolobium* | tree | *Crematogaster mimosae* |
| *Acacia drepanolobium* | tree | *Tetraponera penzigi* |
| *Larix occidentalis* | tree | *Suillus spectabilis* |
| *Larix occidentalis* | tree | *Suillus grisellus* |
| *Larix occidentalis* | tree | *Suillus grevillei* |
| *Parasponia andersonii* | tree | *Bradyrhizobium spp.* |
| Unicellular eukaryotic hosts | | |
| *Paramecium bursaria* | Paramecium | *Chlorella spp.* |
| *Dictyostelium discoideum* | amoeba | *Burkholderia hayleyella* |
| *Dictyostelium discoideum* | amoeba | *Burkholderia agricolaris* |

**Table S2.** Closest relative to symbiont species included in meta-analysis. We built the symbiont phylogeny using the closest relative, then replaced the relative on the tree with the study species.

| **Original species** | **Closest relative found in OTL** |
| --- | --- |
| Acetobacteraceae spp. | *Acidisoma* |
| *Aquicella* spp. | Coxiellaceae |
| *Breviolum muscatinei* | *Breviolum psygmophilum* |
| *Commensalibacter intestini* | *Acetobacter pomorum* |
| *Desulfovibrio* spp. | Desulfovibrionaceae |
| Lactobacillaceae spp. | *Convivina* |
| *Nardonella* spp. | *Aeromonas* |
| Porphyromonadaceae spp. | *Microbacter* |
| Ruminococcaceae spp. | *Flavonifractor* |
| *Sodalis pierantonius* | *Sodalis melophagi* |

**Table S3**. Results of overall and moderator analyses, with all effect sizes or with outliers removed.

| **OVERALL** | | | | | | |
| --- | --- | --- | --- | --- | --- | --- |
| **analysis** | **estimate** | **SE** | **zval** | **pval** | **CI (upper)** | **CI (lower)** |
| host association | -1.773  (all effect sizes) | 2.828 | -0.627 | 0.531 | -7.315 | 3.769 |
| host association | -1.744  (1 outlier removed) | 2.864 | -0.609 | 0.542 | -7.358 | 3.868 |
| environment | -1.011  (all effect sizes) | 1.987 | -0.509 | 0.611 | -4.906 | 2.884 |
| environment | -0.701  (5 outliers removed) | 2.045 | 0.343 | 0.732 | -4.708 | 3.3066 |
| time | -3.354  (all effect sizes) | 1.329 | -2.524 | 0.012 | -5.958 | -0.750 |
| time | -2.324  (5 outliers removed) | 1.085 | 2.142 | 0.032 | -4.451 | -0.198 |
|  | | | | | | |
| **MODERATORS** | | | | | | |
| **analysis** | **moderator** | **QM** | **df** | **pval** | | |
| host association | host kingdom  (all effect sizes) | 0.755 | 2 | 0.686 | | |
| host association | host kingdom  (10 outliers removed) | 0.767 | 2 | 0.682 | | |
| host association | type of association | 6.809 | 3 | 0.078 | | |
| host association | type of association  (3 outliers removed) | 6.469 | 3 | 0.091 | | |
| host association | symbiont dependence on host | 3.211 | 1 | 0.073 | | |
| host association | symbiont dependence on host  (4 outliers removed) | 2.910 | 1 | 0.088 | | |
| host association | symbiont diversity | 0.027 | 1 | 0.871 | | |
| host association | symbiont diversity  (2 outliers removed) | 0.0092 | 2 | 0.923 | | |
| host association | host reproductive mode | 0.0457 | 1 | 0.831 | | |
| host association | host reproductive mode  (1 outliers removed) | 0.061 | 1 | 0.805 | | |
| host association | location of symbiont | 0.046 | 1 | 0.831 | | |
| host association | location of symbiont  (1 outliers removed) | 0.061 | 1 | 0.805 | | |
| host association | genome size | 0.407 | 1 | 0.524 | | |
| host association | genome size  (1 outliers removed) | 0.426 | 1 | 0.514 | | |
| environment | host kingdom | 2.899 | 2 | 0.235 | | |
| environment | host kingdom  (9 outliers removed) | 7.2993 | 2 | 0.026 | | |
| environment | type of association | 2.124 | 3 | 0.547 | | |
| environment | type of association  (3 outliers removed) | 2.256 | 3 | 0.520 | | |
| environment | symbiont dependence on host | 0.025 | 1 | 0.874 | | |
| environment | symbiont dependence on host  (10 outliers removed) | 0.7495 | 1 | 0.387 | | |
| environment | symbiont diversity | 0.3577 | 2 | 0.8362 | | |
| environment | symbiont diversity  (8 outliers removed) | 1.961 | 2 | 0.375 | | |
| environment | host reproductive mode | 0.985 | 1 | 0.321 | | |
| environment | host reproductive mode  (7 outliers removed) | 1.055 | 1 | 0.304 | | |
| environment | location of symbiont | 0.402 | 2 | 0.818 | | |
| environment | location of symbiont  (14 outliers removed) | 0.132 | 2 | 0.936 | | |
| environment | genome size | 0.641 | 1 | 0.423 | | |
| environment | genome size  (6 outliers removed) | 0.547 | 1 | 0.547 | | |
| environment | host kingdom  (intracellular symbionts only) | 4.224 | 2 | 0.121 | | |
| environment | host kingdom  (intracellular symbionts only)  (7 outliers removed) | 12.246 | 2 | 0.002 | | |
| environment | host generation time for unique host-symbiont pairings | 0.0588 | 1 | 0.8084 | | |
| environment | host generation time for unique symbionts | 5.036 | 1 | 0.025 | | |
| environment | symbiont genome size for unique symbionts | 4.524 | 1 | 0.033 | | |
| time | host kingdom | 3.539 | 2 | 0.170 | | |
| time | host kingdom  (1 outliers removed) | 3.610 | 2 | 0.165 | | |
| time | type of association | 0.235 | 3 | 0.972 | | |
| time | type of association  (4 outliers removed) | 1.864 | 3 | 0.601 | | |
| time | symbiont dependence on host | 0.2125 | 1 | 0.2125 | | |
| time | symbiont dependence on host  (4 outliers removed) | 1.310 | 1 | 0.252 | | |
| time | symbiont diversity | 2.467 | 2 | 0.291 | | |
| time | symbiont diversity  (2 outliers removed) | 2.423 | 2 | 0.298 | | |
| time | host reproductive mode | 3.569 | 3 | 0.312 | | |
| time | host reproductive mode  (1 outliers removed) | 3.617 | 3 | 0.306 | | |
| time | location of symbiont | 8.609 | 2 | 0.014 | | |
| time | location of symbiont  (3 outliers removed) | 9.2153 | 2 | 0.010 | | |
| time | host life stage | 6.235 | 2 | 0.044 | | |
| time | host life stage  (2 outliers removed) | 6.284 | 2 | 0.043 | | |
| time | genome size | 0.006 | 1 | 0.9400 | | |
| time | genome size  (2 outliers removed) | 0.0003 | 1 | 0.986 | | |
